# Small molecule screening platform for assessment of cardiovascular toxicity on adult zebrafish heart

**DOI:** 10.1186/1472-6793-12-3

**Published:** 2012-03-26

**Authors:** Satish Srinivas Kitambi, Erik S Nilsson, Petra Sekyrova, Cristian Ibarra, Gilbert Nyah Tekeoh, Michael Andäng, Patrik Ernfors, Per Uhlén

**Affiliations:** 1 Department of Medical Biochemistry and Biophysics, Division of Molecular Neurobiology, Karolinska Institutet, Stockholm 17177, Sweden

**Keywords:** Heart, Screening, Zebrafish, Small molecule, *Ex vivo*, Ca^2+ ^signaling

## Abstract

**Background:**

Cardiovascular toxicity is a major limiting factor in drug development and requires multiple cost-effective models to perform toxicological evaluation. Zebrafish is an excellent model for many developmental, toxicological and regenerative studies. Using approaches like morpholino knockdown and electrocardiogram, researchers have demonstrated physiological and functional similarities between zebrafish heart and human heart. The close resemblance of the genetic cascade governing heart development in zebrafish to that of humans has propelled the zebrafish system as a cost-effective model to conduct various genetic and pharmacological screens on developing embryos and larvae. The current report describes a methodology for rapid isolation of adult zebrafish heart, maintenance *ex vivo*, and a setup to perform quick small molecule throughput screening, including an in-house implemented analysis script.

**Results:**

Adult zebrafish were anesthetized and after rapid decapitation the hearts were isolated. The short time required for isolation of hearts allows dissection of multiple fishes, thereby obtaining a large sample size. The simple protocol for *ex vivo *culture allowed maintaining the beating heart for several days. The in-house developed script and spectral analyses allowed the readouts to be presented either in time domain or in frequency domain. Taken together, the current report offers an efficient platform for performing cardiac drug testing and pharmacological screens.

**Conclusion:**

The new methodology presents a fast, cost-effective, sensitive and reliable method for performing small molecule screening. The variety of readouts that can be obtained along with the in-house developed analyses script offers a powerful setup for performing cardiac toxicity evaluation by researchers from both academics and industry.

## Background

The drug discovery process is heavily impeded by cardiovascular toxicity [1]. The sooner toxicity is discovered, the better for preclinical safety standards and cost of drug development. Various *in vitro *assays such as membrane potential dyes [2], rubidium efflux [3], radioligand binding [4] and patch clamp [5] have been used to identify compounds displaying cardiac toxicity. Since a compound can affect more than one target, it is necessary to understand the effects on the whole organ system. Therefore, various *ex vivo *assays such as the purkinje fiber assay [6], isolated heart assay [7] and *in vivo *assay using transgenic mice [8] have emerged. However, these assays are limited in specificity, reliability and throughput efficiency. Thus, more model systems are needed to evaluate cardiovascular toxicity.

Zebrafish has gained immense popularity as a model for small molecule screening for factors influencing the cardiovascular system [9-11]. Various screens have been performed on zebrafish embryos and larvae to study factors influencing development and homeostasis of the cardiovascular system [11]. The availability of transgenic zebrafish lines expressing fluorescent proteins in the vascular system, blood, endothelial cells and heart, facilitates precise and accurate evaluation of the effects of compounds on the cardiovascular system [12]. The small size, relative ease in handling, and the possibility of obtaining a large number of samples (embryos or adult fish) for statistical evaluation, offers a reliable platform for performing small molecule screening.

The current report describes a method for rapid isolation of intact adult zebrafish hearts, maintenance *ex vivo *and presents a platform for small molecule screening on multiple hearts. This setup offers a reasonable throughput-screening platform for generating rapid and statistically significant readouts. Readouts can range from recording heartbeats or temporal changes in morphology to measuring cellular calcium (Ca^2+^) signaling, rhythmic beating and force of contraction. Taken together, this *ex vivo *cardiac assay model offers a powerful tool for evaluating several dimensions of potential cardiovascular toxicity of lead compounds.

## Methods

### Preparation of reagents

Adult wild type AB strain zebrafish to be dissected were transferred to Egg Water containing "Instant Ocean" sea salts to a final concentration of 60 mg/ml, and anesthetic solution containing 0.1% Tricaine (Sigma) in Egg Water was prepared as described in *The Zebrafish Book *(University of Oregon Press, 2000). Krebs-Ringer's solution (onward referred to as Krebs solution) for dissection and screening was prepared by mixing NaCl (119.0 mM), KCl (2.5 mM), NaH_2_PO_4 _(1.0 mM), CaCl_2_: 2H_2_O (2.5 mM), MgCl_2_: 6H_2_O (1.3 mM), HEPES (20 mM) and D-glucose (11.0 mM). The solution was adjusted to pH 7.4 and filter sterilized using a 0.2 μm filter (Sarstedt). If Ca^2+ ^responses were to be recorded, Fura-2/AM (Molecular Probes) was used at a final concentration of 0.2 mM in Krebs solution. Culture medium for maintaining the hearts was prepared by adding fetal bovine serum (Invitrogen) to a final concentration of 10% in DMEM (Invitrogen). The 0.5 to 1% low-melt agarose solution was prepared in filtered Krebs solution.

### Animal husbandry and rapid dissection of the adult zebrafish heart

**Figure 1:**
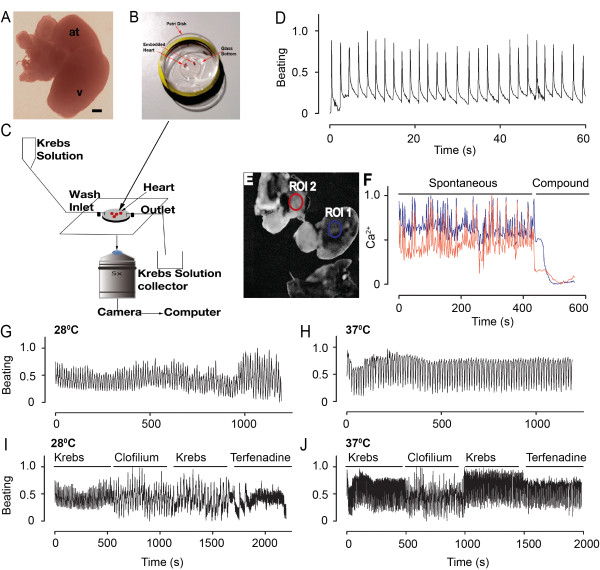
**A) Whole heart, isolated from wild type adult zebrafish showing Atrium (abbreviated: at) and Ventricle (abbreviated: v)**. B) Petri dish with embedded hearts used for screening. C) Diagrammatical representation of the screening setup. Petri dish with embedded hearts is placed on an inverted microscope having an in- and out-let to regulate the in- and out-flow of fresh Krebs buffer. D) Analyses of heartbeating using MATLAB. E) Three hearts mounted on a Petri dish to measure Ca^2+ ^responses. The regions of interest (ROI1 and ROI2) that were used to analyze Ca^2+ ^responses are shown with blue and red circles. F) Graph showing spontaneous Ca^2+ ^activity and compound-induced Ca^2+ ^response in ROI1 (in blue) and ROI2 (in red). Analyses of heartbeats at 28°C G) and 37°C H). Analyses of heartbeats obtained from screen of compounds as indicated carried out at 28°C I) and 37°C J). Compound concentrations of 10 μM were used in the screen. Scale bar: 200 μm in A.

Adult zebrafish of 4-6 months were anesthetized and immediately transferred to a Petri dish under a microscope for dissection. Using two forceps (Dumont Forcep No 5, Sigma), the zebrafish was rapidly decapitated. One forceps was used to hold the body of the zebrafish and the other forceps was then inserted into the body cavity below the pectoral fin and the overlying skin was pulled away to reveal the beating heart. The heart was carefully pulled away from the rest of the body and all other tissues were cleared from the Petri dish (Figure 1A).

### *Ex-vivo *culture

The hearts were transferred to a 12 well plate, each well having 1 ml of culture medium, and incubated at 37°C. We have successfully maintained beating hearts for up to 4 days without any contamination. Maintaining the hearts at temperature optimal for fish (28.3°C) can also be done without major impact on its survival and performance. For evaluating this assay, organotypic culture of adult mice heart and *in vitro *culture of immortalized HL-1 cardiomyocytes were done using previously described protocols [13,14]

### Setup for performing small molecule screening

The screening procedure can be performed with 3-5 heart preparations, either embedded or un-embedded in agarose, in a glass bottom Petri dish (MatTek). First, the glass bottom of the dish was coated with laminin (Sigma), diluted 1:200 with Phosphate Buffered Saline (Invitrogen) from the stock concentration of 1 mg/ml and incubated for at least 3 h at 37°C. Excess laminin solution was removed and 3-5 hearts were transferred to the Petri dish with as little culture medium as possible. If Ca^2+ ^measurements were to be performed, the hearts were incubated for 10 min at 37°C with 5% CO_2_, immersed in 100 μl of 0.5 mM of Fura-2/AM. Next, 150 μl of Krebs solution was added to the Petri dish and incubated for an additional 30 min. After transfer of the heart to the Petri dish, excess culture medium or Fura-2/AM was removed and 1-2 drops of low melt agarose (Lonza) was pipetted onto the heart (Figure 1B). The agarose was allowed to solidify and 1 ml of pre-warmed Krebs solution was added into the Petri dish. The same setup was also used for performing comparative screens on mouse heart and HL-1 cell cultures.

### Performing screening on zebrafish heart

**Figure 2:**
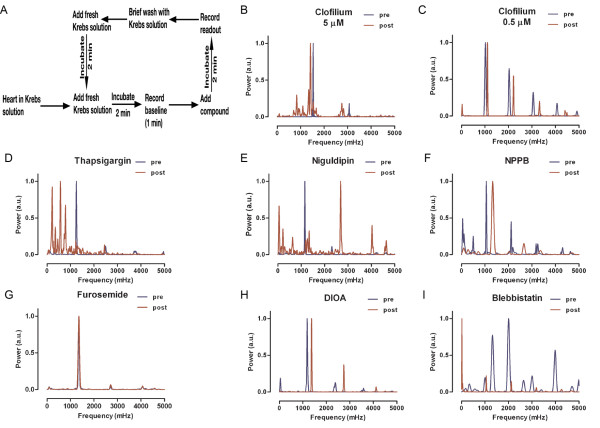
**A) Flowchart showing the screening procedure to assess compounds**. B-I) Spectral analyses of heartbeats from hearts treated with various compounds. Spectra from control (DMSO) treatments or untreated hearts are shown in blue, while spectra from compound exposures are shown in red. B-C) Spectra from hearts treated with two different concentrations, as indicated, of clofilium. Spectra from hearts treated with different Ca^2+ ^channel modulators D-E), chloride transport inhibitors F-H) and blebbistatin I). Compound concentrations of 10 μM were used in the screen. Data shown in panels B-D, E-G and H-L were generated by three separate screening.

The Petri dish with embedded hearts was moved to the screening station (Figure 1C) and mounted on a temperature-controlled stage setup (Warner Instruments) clamped onto a Zeiss Axiovert 100 M microscope, equipped with a 25X/0.8NA water immersion objective and a 5x/0.15NA objective (all from Carl Zeiss), connected to a Lambda LS xenon-arc lamp, Lambda 10-3 filter-wheel and a *smart*Shutter (all from Sutter Instruments). A container was filled with freshly prepared and pre-warmed Krebs solution for washing the samples in between compound treatments. A peristaltic pump was used to add and remove the washing buffer to hearts in the mounted Petri dish. Recording of heartbeats (Figure 1D) or spontaneous Ca^2+ ^signals (Figure 1E-F) were acquired at 5 and 0.5 Hz respectively with an EMCCD camera Cascade II:512 (Photometrics) controlled by the acquisition software MetaFluor (Molecular Devices). A control reading was acquired before starting screening. Compounds were then added directly to the Petri dish using a pipette and changes in heart beating or Ca^2+ ^signaling were captured. Following this procedure, the compound was rinsed off by allowing Krebs solution to flush the hearts. Thereafter, the recording continued until a signal was detected that resembled the initial control reading whereupon a second compound was added (Figure 2A). This process could be repeated as long as a stable control reading could be detected after each treatment.

### Quick analyses of the heartbeats

Bright field time-lapse movies of beating hearts were imported to ImageJ and regions of interest (ROI) encompassing the border of the hearts were marked. The mean intensities of each time point in the time-lapse recording were extracted, using the MultiMeasure plugin, and saved in a .*dat *file. The values within this file were thereafter imported to an in-house MATLAB software tool beating.m (Additional file 1: Table S1) or into a previously described spectral analysis software tool, also implemented in MATLAB, to characterize the temporal properties of heart beating [15]. These algorithms normalize the time-series and remove any possible trend-lines, produced by e.g. focus shift, by fitting the trace to a polynomial (Figure 1D).

Single or multiple heart preparations were used for capturing dynamics of Ca^2+ ^signaling (Figure 1E). For analyzing Ca^2+ ^signals, the MetaFluor software was used to extract the Fura-2/AM intensities in different ROIs. The data was then exported to Excel and a graph was plotted (Figure 1F).

## Results and discussion

In order to set up the screening platform for dissected hearts, real-time recordings of heart beating (Figure 1D) and Ca^2+ ^fluxes (Figure 1E-F) were performed and analyzed using an in-house developed algorithm implemented in MATLAB (Additional file 1: Table S1). Both heartbeats and Ca^2+ ^recordings displayed steady periodicity with little fluctuations over time. As the heartbeat recordings were more robust and easy to conduct, this readout was used in further screens. To validate the screening platform, the beating of the heart was monitored at both 28°C and 37°C (Figure 1G-H), following application of three hERG channel modulators, using a screening flowchart (Figure 2A). The beating and response to compounds were more significant at 37°C than at 28°C (Figure 1I-J). In drug development, human hERG potassium channels are of utmost importance as they are crucial for cardiac action potential repolarization. Inhibition or reduced function of hERG channels delay ventricular action potentials causing cardiac arrhythmia which can result in cardiac arrest. Hence, it is of utmost importance that hERG related effects of novel compounds are identified at early stage of drug development. Similar to hERG-induced cardiac arrhythmia in humans, a clear impact on the amplitude and frequency of beating was seen when compounds were administered to the zebrafish heart. This indicated that screening could be reliably performed at 37°C, using both acute and chronic assessments to carry out real-time recordings to provide additional mechanistic insights into the effect of compounds on cardiac activity. Our data also indicates that this model offers a good platform to test hERG-related effects of compounds.

To evaluate beating irregularities, spectral analysis was utilized on the time-series from beating hearts. The Fourier transform converts the signal in time-domain to its correspondence in frequency domain. The peaks in the spectra represent different frequencies of sine wave components of the original oscillatory signal. Compounds producing spectra identical to untreated hearts were considered to have no cardiac effect. Dramatic changes in heart beating (as seen in Additional file 2: Movie S2 showing the effect by Clofilium when compared to no effect of DMSO control in Additional file 3: Movie S3) and shifts in spectra were clearly observed with clofilium (Figure 2B), a potent hERG channel inhibitor. Clofilium at concentrations below 5 μM produced spectra similar to untreated hearts, indicating lack of readable effects (Figure 2C-D). As expected, modulators of Ca^2+ ^signaling were producing striking shifts in the spectra (Figure 2E-F). Compounds modulating potassium channels (Figure 2B-D) and inhibitors of chloride channels had less effects (Figure 2H-J). The inhibitor of myosin heavy chain ATPase activity, blebbistatin, had a dramatic effect on the spectrum (Figure 2K). Galantamine, a previously used Alzheimer's disease drug, with no documented cardiac effects, was without effect on the spectrum (data not shown), indicating no cardiac toxicity. Taken together, these results demonstrated that this setup offers a reliable platform for drug screening.

**Figure 3:**
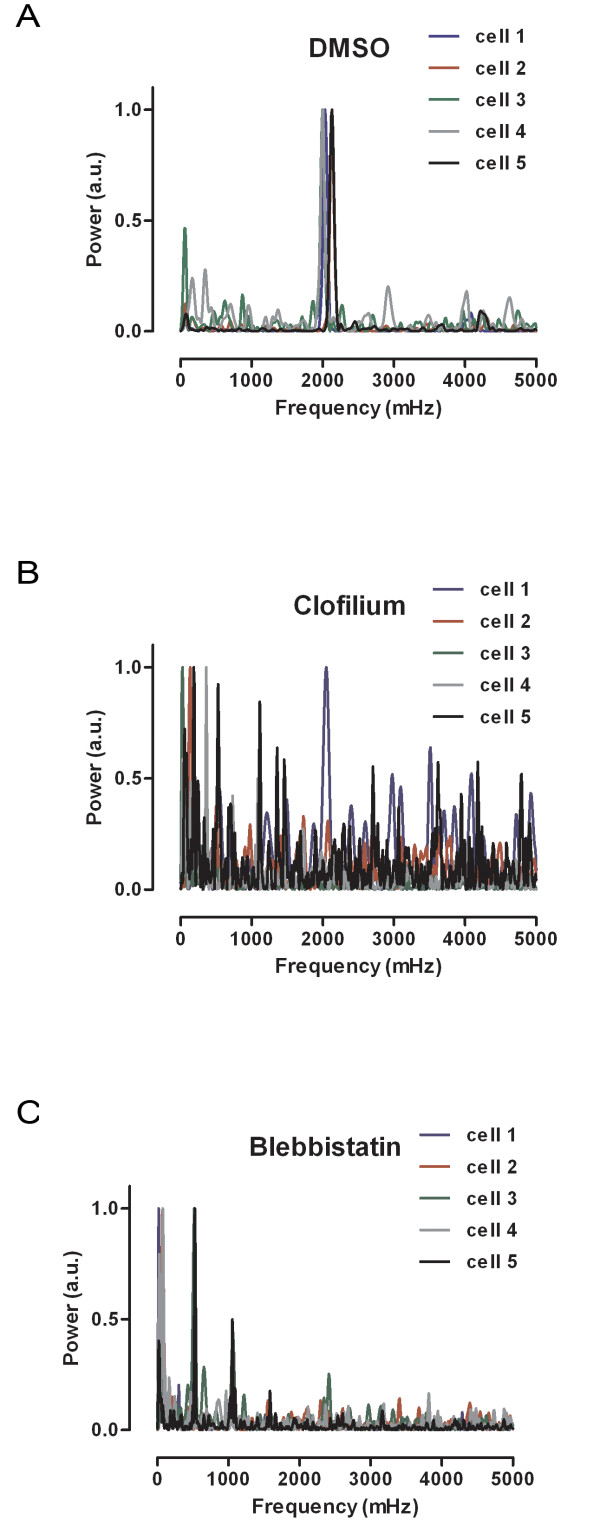
**Spectral analyses of the beating efficiency of immortalized HL-1 cardiomyocytes after exposure to DMSO (A), clofilium (B) and blebbistatin (C)**. Compound concentrations of 1 μM were used in the screen.

Similar experiments on organotypic cultures of mouse heart sections and on cultured cardiomyocytes were carried out for comparison. Exposure of cardiomyocytes to clofilium or blebbistatin yielded similar spectrums as produced by the zebrafish heart (Figure 3A-C). When clofilium was applied, the beating of the mouse heart ceased (data not shown). Our data shows that this assay can sensitively and precisely detect a range of effects that complement existing models. Thus, this assay has the potential to be used as a major screening model system for cardiac toxicity assessment or can be integrated as a part of multi-model cardiac toxicity risk assessment.

Small molecule screening of compounds affecting cardiovascular performance in zebrafish embryo or larvae has been immensely successful thereby reflecting on the robustness of this model as a reliable screening platform. Accumulating data indicate a very high degree of developmental and functional similarity between zebrafish and human heart, facilitating evaluation of cardiac-toxicity in terms of human health risks. This platform allows usage of multiple hearts for testing/screening with generation of statistically significant data and little sample variation. The isolated hearts can be maintained in culture *ex vivo *and screening can be performed with multiple candidate molecules, with alternating washing steps. The time required for assessment of one compound is around 3-5 min, allowing scalability and automation. The number of compounds that can be used in one screen depends on whether a stable baseline recording is established after the compound is washed away. The possibility of maintaining beating hearts *ex vivo *allows recording of acute and chronic effects on the cardiac function and viability. Short-term or long-term readouts like heartbeat, mechanical contraction, viability, hERG activity, or Ca^2+ ^fluxes are easy and straightforward to measure, thereby offering a sensitive model to assess cardiac toxicity. Screening with zebrafish heart maintained *ex-vivo *for four days yield similar results as screening on freshly isolated heart (data not shown), however, the number of compounds that can be tested with a single heart markedly decreased (data not shown). Compounds primarily affecting the contractility properties of the heart (eg. Blebbistatin) may be missed by electrophysiological methods, but can be evaluated here. Although this model requires extensive validation, it clearly demonstrates features such as sensitivity to generated readouts, reliability and adaptability with other available methods (such as *in vitro *cardiomyocyte screens). The Matlab script and the spectral analyses allow display of the readout in time domain or in frequency domain respectively, thereby providing deeper understanding of the effects produced by the compounds.

Cardiac safety evaluation is an important part of the drug discovery and development process; hence a whole organ assay model would be highly beneficial. Numerous drug candidates are regularly evaluated using multiple cell lines based assays. Although the adult heart-screening assay requires higher concentrations of test compounds than cell lines or zebrafish embryos/larvae, this assay offers a possibility to test various aspects on whole organ from an adult fish. Since diverse cell types contribute to the function of the entire adult heart, this assay allows precise measurements of the effects produced on all type of cells, governing the function of the organ. Rescue screens performed on hearts isolated from adult mutant zebrafish with heart defects (eg. *Slow mo*) will be highly beneficial for studies on heart development, regeneration and homeostasis using chemical genetics, as well as aiding in the process of drug discovery.

## Conclusion

Features such as rapid dissection and maintaining the heart *ex vivo *for several days offers an excellent possibility for carrying out time series recording or acute and chronic studies in addition to compound screens. The small size and relatively cheap housing of the fish provides a cost effective setup in comparison to other *in vivo *or *ex vivo *models. The hearts can be easily processed for immunohistochemistry or *in situ *hybridization with standard protocols thereby enabling testing of the expression of various biomarkers before and after exposure to the test compounds. Experiments aiming at determining the structure activity relationship (SAR) or reverse medicinal chemistry approach to classify the effect of various structurally diverse compounds on the heartbeat or the spectral profile it generates can be easily adapted to this model. In conclusion the current report clearly demonstrates a platform for performing cardiac toxicity evaluation either as a primary screen or as complement to existing methodologies.

## Authors' contributions

SK conceived the study, SK and EN designed the work, SK, EN, PS, CI, GNT carried out the experiments, MA, PE, PU provided material support and constructive analyses of the data. All authors contributed to writing the manuscript, analysis of the results and approved the manuscript.

## Supplementary Material

Additional file 1 **Table S1**. MATLAB script for analyzing heartbeats. Click here for file

Additional file 2 **Movie S2**. Movie of beating zebrafish heart after addition of Krebs buffer containing clofilium to a final concentration of 10 μM. Click here for file

Additional file 3 **Movie S3**. Representative movie showing beating of zebrafish heart in Krebs buffer and after addition of DMSO control. Click here for file
