# Supplementary material for: Small molecule screening platform for assessment of cardiovascular toxicity on adult zebrafish heart
Source: BMC Physiol. 2012 Mar 26;12:3. doi: 10.1186/1472-6793-12-3 (PMC3334682; doi:10.1186/1472-6793-12-3)
Supplement: Additional file 1 — Table S1. MATLAB script for analyzing heartbeats. [file 1472-6793-12-3-S1.DOC]

%Sampling time

sampling=5;

%Select directory for beating data

[file_name, path_name] = uigetfile({'*.dat';'*.*'},'Select beating data','C:');

if file_name==0

disp('Error! No chosen directory!')

end

fid1 = fopen([path_name, file_name]);

%Remove titles etc.

fid2 = fopen('temp.dat','w'); %Temp file

i=0;

while ~feof(fid1)

n=fgetl(fid1);

if length(find(n=='M'))>0, i=i+1; end

if length(find(n=='M'))==0, fprintf(fid2,[n '\n']); end %Put line without M in temp file

end

fclose(fid2);

fclose(fid1);

%Load data

load temp.dat

beating_data=temp;

delete('temp.dat')

time=beating_data(:,1);

time=time/sampling;

[N,M]=size(beating_data);

number_of_hearts=M-1;

y=beating_data(:,2:M);

for i=1:number_of_hearts

%Remove polynomial trend

poly_fit=polyfit(time,y(:,i),3);

y(:,i)=y(:,i)-polyval(poly_fit,time);

%Normalise amplitude

min_value=min(y(:,i));

y(:,i)=y(:,i)-min_value;

max_value=max(y(:,i));

y(:,i)=y(:,i)/max_value;

end

%Plot beating

plot(time,y)

%title('Beating of dissected zebra fish heart')

%ylabel('Amplitude (a u)')

%xlabel('Time (s)')

**Table S1: MATLAB script for analyzing heartbeats**
